# The β-blocker carvedilol and the benznidazole modulate the cardiac immune response in the acute infection induced by Colombian strain of the *Trypanosoma cruzi*

**DOI:** 10.1590/0074-02760180271

**Published:** 2018-10-18

**Authors:** Aline Luciano Horta, Vivian Paulino Figueiredo, Ana Luisa Junqueira Leite, Guilherme de Paula Costa, Ana Paula de Jesus Menezes, Camila de Oliveira Ramos, Tamiles Caroline Fernandes Pedrosa, Frank Silva Bezerra, Paula Melo de Abreu Vieira, André Talvani

**Affiliations:** 1 Universidade Federal de Ouro Preto, Departamento de Ciências Biológicas, Ouro Preto, MG, Brasil; 2 Universidade Federal de Ouro Preto, Programa de Pós-Graduação em Ciências Biológicas, Ouro Preto, MG, Brasil; 3 Universidade Federal de Ouro Preto, Programa de Pós-Graduação em Saúde e Nutrição, Ouro Preto, MG, Brasil; 4 Universidade Federal de Ouro Preto, Programa de Pós-Graduação em Biomas Tropicais, Ouro Preto, MG, Brasil

**Keywords:** carvedilol, Trypanosoma cruzi, inflammation, heart disease, chemokines

## Abstract

BACKGROUND The infection led by *Trypanosoma cruzi* persists in mammalian tissues causing an inflammatory imbalance. Carvedilol (Cv), a non-selective beta blocker drug indicated to treat heart failure and antihypertensive has shown to promote antioxidant and immunomodulatory properties which might improve the inflammation induced by *T. cruzi*. OBJECTIVES Evaluate the role of Cv on the inflammatory response of C57BL/6 mice acutely infected with the Colombian strain of *T. cruzi*. METHODS Animals were infected with the Colombian strain of *T. cruzi* and treated with Cv (25 mg/kg/day), benznidazole (Bz) (100 mg/kg/day) or their combination. On the 28th day of infection and 23 days of treatment, the euthanasia occurred, and the heart preserved for histopathological, oxidative stress (SOD, catalase, TBARs, carbonylated proteins) and plasma (CCL2, CCL5, TNF, IL-10) analyses. Parasitaemia and survival were assessed along the infection. FINDINGS Cv decreased TBARs, but increased the mortality rate, the parasitaemia and the levels of CCL2, CCL5, catalase and the inflammatory infiltrate in the cardiac tissue. Bz led the reduction of the inflammatory infiltrate and circulating levels of oxidative stress and inflammatory mediators in the infected mice. MAIN CONCLUSIONS Our data suggest that Cv, in this experimental model using the Colombian strain of *T. cruzi*, caused damage to the host.

Inflammation is one of the oldest medical conditions registered. It was described in the first century AC with four main signs - red, heat, swelling, pain - by the Roman academician Aulus Cornelius Celsus. Nowadays, inflammation can be defined as an adaptive response to tissue malfunction or homeostatic imbalance, known to perform an important role in host defense against potential threats.^1^

Protozoa, such as *Trypanosoma cruzi*, can elicit an acute inflammatory response through its surface molecules, releasing a great number of soluble mediators that includes cytokines, chemokines, oxygen derivatives, amines, lipids and others. This parasitic infection causes an illness known as Chagas disease, responsible for a great number of mortality rate, affecting nearly 6 million people worldwide, mostly in Latin America countries.^2^ Cardiac involvement is the most severe manifestation of Chagas disease, and although immune response is essential for host defense, it behaves as a double-edged sword, where the persistent inflammation induced by this protozoan is crucial for the onset and progression of this condition, entailing the destruction of cardiomyocytes and culminating in myocarditis, fibrosis and changes in the heart architecture and functionality.^3^ Oxidative stress is one of the main weapons of inflammation to face parasitic infections. However, some studies have shown that the increase of reactive oxygen species (ROS) in cardiac tissue in response to *T. cruzi* infection, is driven by cytokines and chemokines, which enhances the oxidation of indispensable cell components, compromising its structure, function, and consequently resulting in cell death.^4^ ^,^ ^5^

Current treatments regarding this disease is almost exclusively based on antiparasitic chemotherapy [i.e., benznidazole (Bz) and nifurtimox (Nfx)], and despite its effectiveness in the acute phase of this illness, these agents display unsatisfactory results in chronic Chagas disease and high toxicity that leads most patients into therapy abandonment.^6^ For that matter, considering that the sustained inflammatory response coupled with oxidative stress contributes to the pathogenesis of this illness, it would be medically useful the detection of new drugs capable of suppressing this process by blocking these harmful conditions led by inflammation, without undermining host defense. That said, our research group has been evaluating the potential immunomodulatory outcomes of medications routinely managed in clinical chagasic patients. These therapies (simvastatin, enalapril, and doxycycline) perform primarily cardiovascular application, but in experimental models and in humans, they have shown to reduce inflammatory infiltration, fibrosis, and the plasma levels or expression of cytokines and chemokines in experimental models of the *T. cruzi* infection.^7^ ^,^ ^8^

In this proposal, we investigated the role of carvedilol (Cv), a non-selective beta-adrenergic drug, in the cardiac immune response in murine model infected by the Colombian strain of *T. cruzi.*

## SUBJECTS AND METHODS

*Ethics statement* - All procedures described in the current study are in accordance with guidelines issued by the National Council for Control of Animal Experimentation (CONCEA), and this research was previously approved by the Ethics Committee on Animal Research of UFOP - CEUA (Protocol CEUA- No 2016/14).

*Parasite strain* - The Colombian strain of *T. cruzi* was first isolated by blood culture from a chronic patient in Colombia. This strain has been classified as discrete typing units (DTU) I, presenting cardiac tropism and resistant to Bz therapy.^9^ The *T. cruzi* stock was maintained through successive passages in Swiss mice at the Laboratory of Immunobiology of Inflammation (LABIIN), at Federal University of Ouro Preto (UFOP), state of Minas Gerais, Brazil.

**Fig. 1:**
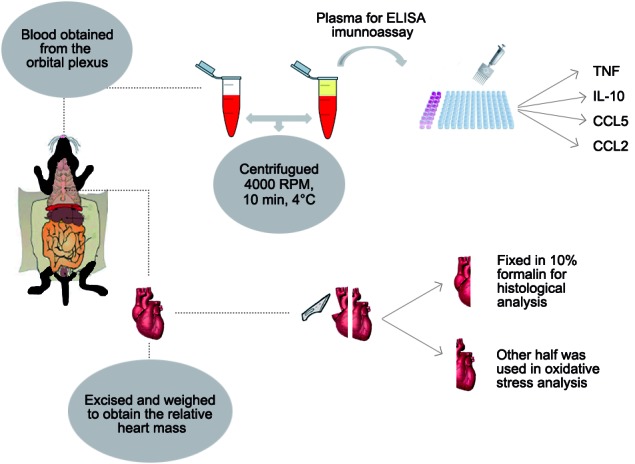
illustrative panel of the trials carried out in this work.

*Experimental animals and infection* - Eight-to ten-week-old Male C57BL/6 mice weighing 18-20g were bred and housed at the Center of Animal Science from UFOP, Brazil. The animals were intraperitoneally inoculated with 50 bloodstream trypomastigote forms of the Colombian strain of *T. cruzi*. Parasite number in blood was determined by daily counting in a fresh blood from tail (5 uL), using an optical microscopy The mice were euthanised after the parasitaemia peak, on the 28th day of infection. Blood was collected for immunoassay, half heart (i) was fixed in 10% formalin for histological analysis, (ii) the other half was used in oxidative stress analysis (Fig. 1).

*Study drugs and treatment scheme* - Cv (±) -1-(carbazoloyl-(4)-oxyl)- 3-(2-methoxy-phenoxy) ethylamino-2-propanol (Coreg®, Roche) was administered orally diluted in PBS, containing methyl cellulose 0.5% for suspension. Bz (2-nitroimidazole-(Nbenzil-2-nitzo-1-imidazoleacetamide, Rochagan, Roche) was used as a reference treatment in this study and was diluted in PBS with the addition of methyl cellulose 0.5% for suspension. The proposed therapies were performed orally by gavage.

The experimental design consisted in 65 inbred male C57BL/6 mice, divided into five treatment groups: (i) 15 mice infected with *T. cruzi* receiving vehicle; (ii) 15 mice infected with *T. cruzi* and treated with Cv (25mg/kg); (iii) 15 mice infected with *T. cruzi* and treated with Bz (100mg/kg); (iv) 15 mice infected with *T. cruzi* and receiving both therapies; and (v) 10 non-infected mice receiving vehicle only, following the same regimen of the animals under the established treatments. All treatment started after five days of infection to guarantee the infection with the protozoan*,* which was confirmed with the parasitaemia curve analysis.

*Heart mass measurement* - To obtain the cardiac hypertrophy index, each animal was weighed before euthanised, and then hearts were carefully excised *ex vivo*, gently dried on absorbent paper and perfused in phosphate saline buffer to remove the excessive blood from the fragments. After this procedure, the organ was weighed on an analytical balance, and the relative heart weight was calculated using the heart weight in mg/mouse body weight in grams (mg/g). The value obtained was then used to evaluate the cardiac mass measurements obtained after 28 days of infection.

*Imunnoassays* - To analyse the interference of Cv therapy on inflammatory response associated with *T. cruzi* experimental infection, immunoassays were performed for inflammatory cytokine and chemokine TNF and CCL2/ MCP-1 (PeproTech, NJ, USA), respectively, and for the regulatory cytokine IL-10 (PeproTech, NJ, USA). Blood from the orbital venous sinus (0.5 mL) was collected during euthanasia and centrifuged (1500 *g* for 15 minutes at 4ºC). The plasma was stored at -80ºC. Next, these samples were used to measure TNF, CCL2, and IL-10, according to the protocol recommended by the manufacturer. The samples were simultaneously measured in triplicate.

*Histopathological analysis* - Cardiac tissue fragments were fixed in 10% buffered-formalin solution, then, they were dehydrated, cleared, and embedded in paraffin, and the blocks were cut in 4mm thick sections and stained by Hematoxylin and Eosin (HE), to quantify the leukocytes infiltration and the amastigote nests. The stained sections were randomly assessed at a 40x magnification, in a total of 74931 µM^2^, the equivalent area of 50 fields of the analysed myocardium. Images were obtained in a Leica DM 5000 B micro chamber (Leica Application Suite, UK, version 2.4.0 R1) and processed in the LeicaQuinn (V3) image analyser software. The inflammatory process was evaluated by counting the number of cellular nuclei found in the infected heart tissue and compared with the number of cardiac cellular nuclei found in the non-infected mice.

Oxidative stress

*Preparation of protein extracts* - Oxidative stress was obtained by the analysis of some enzymes and proteins in homogenised fragments from the cardiac tissue of each animal. Total protein was measured using the Bradford reagent, which contains as a main component the bright blue Coomassie dye, which in acidic solution binds to the proteins of the sample, changing its absorbance from 465 nm to 595 nm and measured by the enzyme-linked immunosorbent assay (ELISA) reader.

*Superoxide dismutase activity (SOD)* - The same cardiac homogenate was used to measure SOD using potassium phosphate monobasic (KH_2_PO_4_), dibasic sodium phosphate (Na_2_HPO_4_), Pyrogallol, MTT [3- (4,5-dimethylthiazol-2H) -2,5-diphenylterazolium bromide] and dimethylsulfoxide (DMSO). Absorbances were read in the ELISA reader at a wavelength of 570 nm.

*Catalase (CAT) activity* - The CAT activity was evaluated by measuring the rate of decomposition of hydrogen peroxide (H_2_O_2_) at an absorbance of 240 nm. Ten microliters of the cardiac homogenate were added to the phosphate buffer and 900 μL H_2_O_2_ (10 mM) and absorbances were read, exactly every minute along 3 min. CAT activity was calculated according to the Lambert Beer rule. The absorbance used in this expression is the delta obtained from the first and last read absorbance (final absorbance - initial absorbance). The molar extinction coefficient used was that of H_2_O_2_ (39.4M -1 cm -1). This concentration was plotted as U/mg protein. One unit of catalase is equivalent to hydrolysis of 1 μmol H_2_O_2_ per min.

*Carbonylated proteins* - To determine the concentration of carbonylated protein the cardiac tissue homogenate was used in potassium phosphate buffer in a concentration of 100 mg/mL, where 0.5 mL of this solution was precipitated with 0.5 mL of 1% trichloroacetic acid (TCA). The supernatant was then discarded and 0.5 mL of dinitrophenyllydrazine in hydrochloric acid (HCl) was added to the pellet so that this system remained in incubated for 1 h with vortex passages every 15 min. Then 0.5 mL of 1% TCA was added and taken to further centrifugation. To the pellet was added 1 mL of ethanol/ethyl acetate 1:1 solution and brought back to the centrifuge. Then 1 mL of 6% of the sodium dodecyl sulfate (SDS) solution was added to the tube and centrifuged. This time the pellet was discarded, and the supernatant was taken for reading in a quartz cuvette spectrophotometer.

*Thiobarbituric acid reactive substances* (*TBARS*) - The determination of the TBARS concentration was underlined on the ability of Thiobarbituric acid (TBA) to bind into oxidised lipids, forming malondialdehyde. The supernatant was removed and used as a biological sample. 500 μL of homogenate, 230 μL of 28% w/v TCA dissolved in 0.23N of HCl, 230 μL of 1% of TBA dissolved in 1:1 acetic acid and 123 μL of 5 mM BHT dissolved in ethanol. The concentration of TBARS was determined using the molar extinction coefficient (ε = 1.56x 105 L x mol-1 x cm -1), following the Lambert Beer rule.

*Statistical analysis* - All analyses were performed using GraphPad Prism v.5 (GraphPad Software, San Diego, CA, USA). Data are expressed as the mean ± standard error of the mean (SEM) and were evaluated by the Kolmogorov-Smirnov test to confirm patterns of normality. Confirming normality, data were analysed by one-way analysis of variance (ANOVA) test for multiple comparisons (Tukey test) and Mann-Whitney test when nonparametric. The level of significance was accepted at p < 0.05.

## RESULTS

**Fig. 2:**
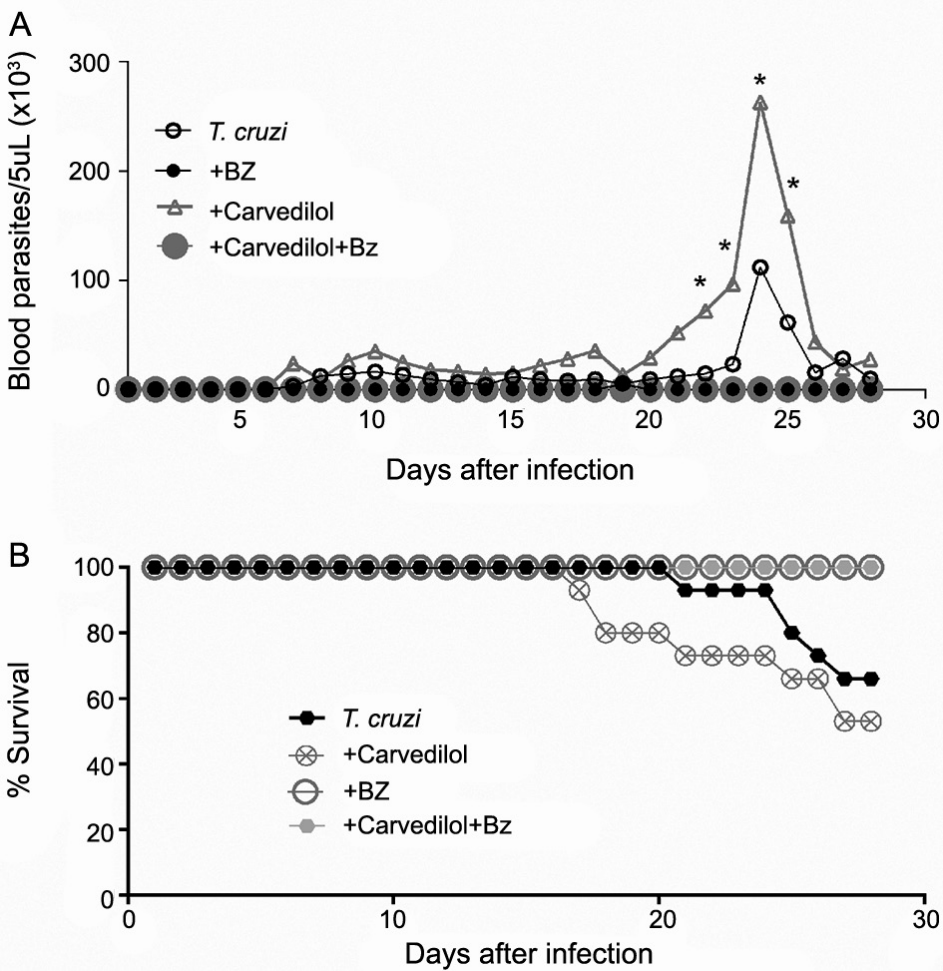
survival and parasitaemia curves. The survival (A) and parasitaemia (B) curves are shown in C57BL/6 mice (n = 15) intraperitoneally infected with 50 blood trypomastigote forms of the Colombian strain of *Trypanosoma cruzi*. The data in each point of the parasitaemia curve represents the average parasitaemia/day for each group. In graphic B, * p < 0,05 means parasitaemia differences in the infected animals treated with carvedilol (Cv) in comparisson to the *T. cruzi*-infected group and those infected treated with benznidazole (Bz) or Cv + Bz.

*Parasitaemia and survival curves* - During the acute phase of *T. cruzi* infection the survival curve was plotted, and among the treated groups, the Bz (100 mg/kg) driven in monotherapy and the combination therapy (Cv + Bz) increased the survival rate in the animals receiving these therapies (Fig. 2A). Whereas, the group of animals that received only the Cv therapy presented approximately 53% of the animals alive by the end of 28 days of infection, exhibiting the lowest survival rate even when compared to the infected animals left untreated.

A similar pattern was observed when analysing the circulating levels of this parasite (Fig. 2B). Besides the effectiveness of both Bz and combination therapy (Cv + Bz) in preventing animals mortality, these therapies, additionally led to parasite clearance in the animal’s bloodstream. However, Cv treatment showed the highest parasitaemia level with significant results when compared to all infected groups treated or not.

**Fig. 3:**
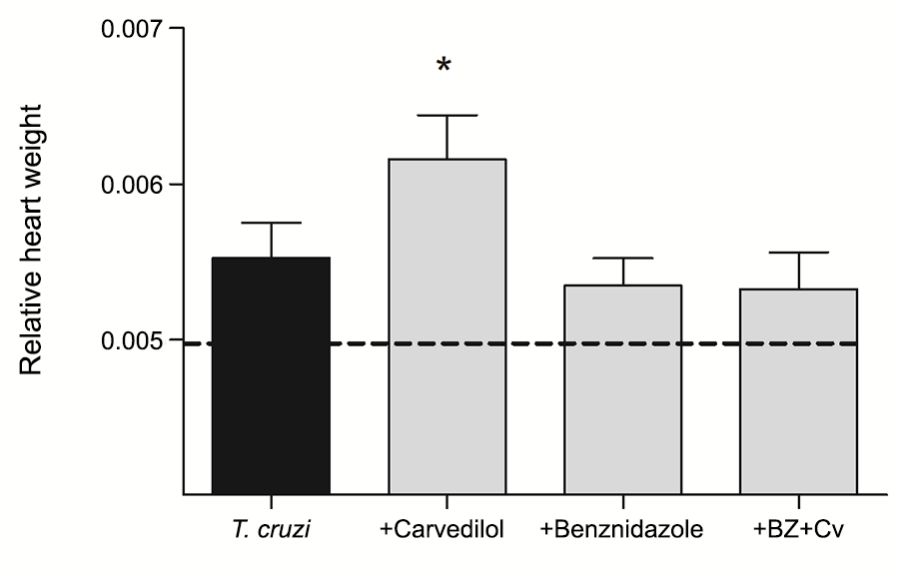
measure obtained by calculating heart mass divided by the animal’s body weight. Relative heart weight of C57BL/6 infected and non-infected (dashed line) with the *Trypanosoma cruzi* Colombian strain and treated (gray bars) or not (black bar) with the carvedilol (Cv) and/or benznidazole (Bz) therapies. * p < 0,05 to the infected animals treated with Cv in comparisson to the *T. cruzi*-infected group and to the Bz or Cv + Bz groups.

*Heart mass measurements* - This measurement was evaluated by dividing the mouse body weight by the heart weight. This measurement was evaluated by dividing the average of the mouse body weight by the heart weight. The untreated infected animals kept heart/body weight ratio similar to the non-infected animals as well as when compared to those treated with Bz and the combining therapy. In contrast, Cv treatment showed an increase in the relative heart mass when compared to the control animals as showed in Fig. 3.

**Fig. 4:**
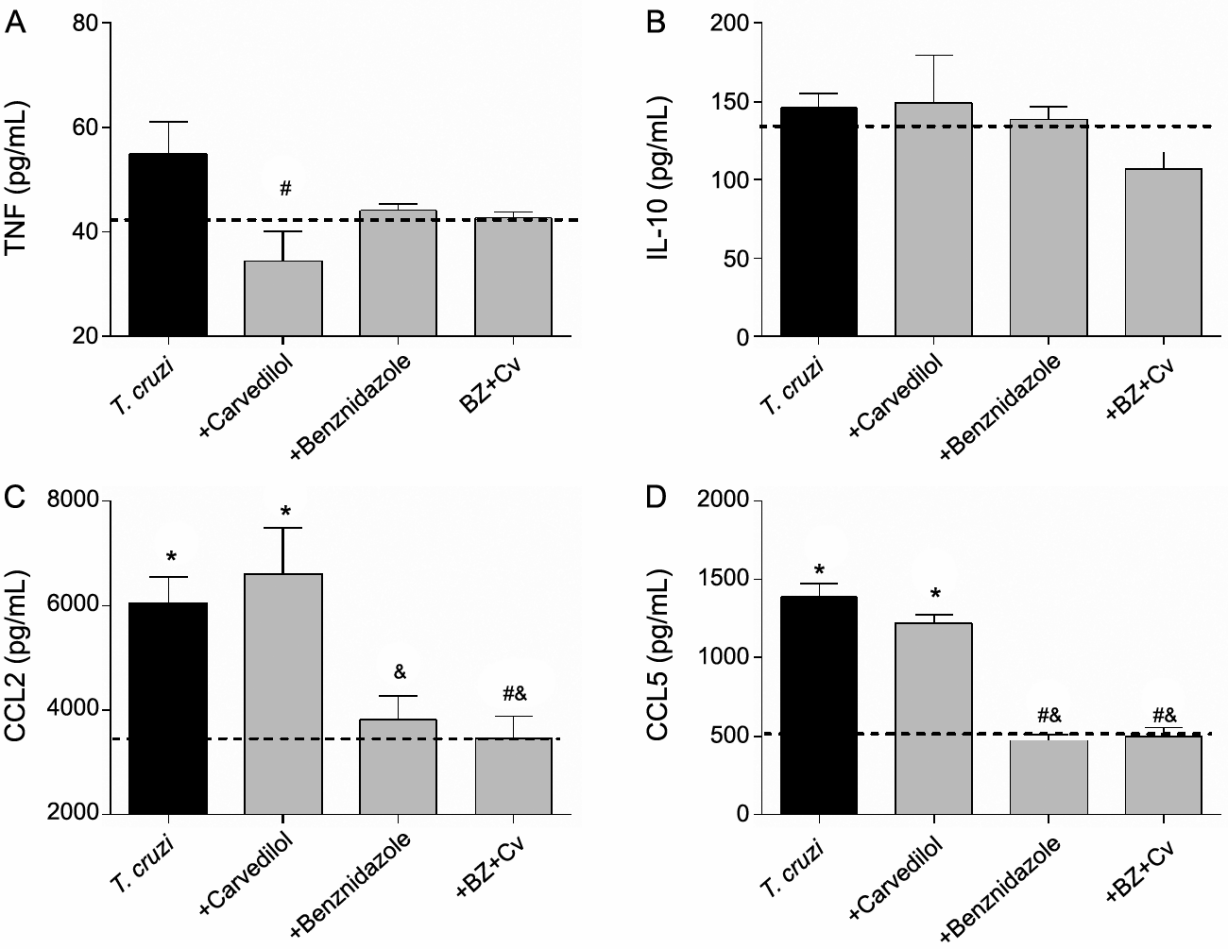
plasma concentration of the cytokines TNF (A), IL-10 (B), and chemokines CCL2 (C) and CCL5 (D) from the plasma of C57BL/6 mice infected with *Trypanosoma cruzi*. Mice were infected or not (dashed line), with the Colombian strain of *T. cruzi*, and were either left untreated (black bar) or treated daily (gray bars) with the proposed therapies. *p < 0.05 when the uninfected group was compared to the infected groups; # p < 0.05 when the infected group was compared with infected groups treated with different drugs; & p < 0.05 when comparing the treatment with carvedilol with the other treatments.

*Inflammatory mediators in the acute phase of experimental infection* - The inflammatory cytokine TNF (Fig. 4A) performs a pivotal role in *T. cruzi* infection. In this study, the plasma levels of TNF reduced in the infected group treated with the Cv in a dose of 25 mg/kg when compared to the infected/untreated group. A similar pattern was observed in the infected groups treated with Bz and the combination therapy.

In parallel, we evaluated the circulating profile of the regulatory cytokine IL-10 (Fig. 4B) and, in contrast to the data concerning the TNF, this cytokine showed no differences among the studied groups. Additionally, chemokines have also been described as pivotal mediators in *T. cruzi* infection, enhancing the recruitment of mononuclear cells into inflamed tissues. Therefore, after evaluating the circulating levels of CCL2 and CCL5, it was noted that the infection was able to significantly elevate the plasma levels of these chemokines and we observed that only Cv was capable to increase the level of the CCL2 while the Bz driven in monotherapy and the combination with Cv were able to reduce the circulating levels of the CCL2 (Fig. 4C) and the CCL5 (Fig. 4D), resembling the levels observed in the control group.

**Fig. 5:**
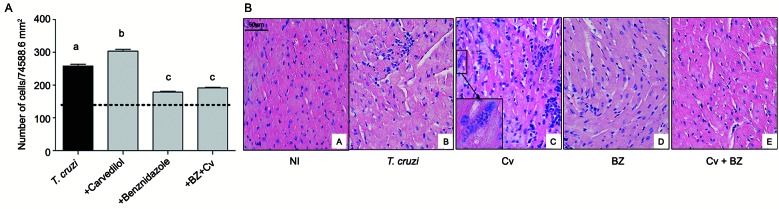
quantification of the acute inflammatory process in the cardiac tissue of mice infected with *Trypanosoma cruzi* Colombian strain. Analysis of inflammatory infiltration in cardiac tissue treated with carvedilol (Cv) and/or Benznidazole (Bz) after 28 days of infection. Bars in black represents the group of infected/untreated animals, bars in gray represents the infected groups under different therapeutic strategies. Dashed line represents the serum concentration of these cytokines/chemokines in uninfected animals (A) and representative imagens are highlighted in (B). *p < 0.05 when the uninfected group was compared to the infected groups; # p < 0.05 when the infected group was compared with infected groups treated with different drugs; & p < 0.05 when comparing the treatment with Cv with the other treatments.

*Inflammatory infiltrate* - The effects of the proposed therapies were also observed on inflammatory infiltration in the cardiac tissue. After 28 days of infection and 23 days of treatment, the untreated infected animals presented a moderate and diffuse inflammatory infiltration showed in the qualitative analysis (Fig. 5A). The Cv in monotherapy showed a significant increase of cellular nuclei in comparison to the other animal groups, depicting an intense and focal inflammatory infiltration. Whereas, both Bz and the combination therapy maintained the number of cellular nuclei similar to those uninfected animals. However, the combination treatment presented a small increase of cellular nuclei.

**Fig. 6:**
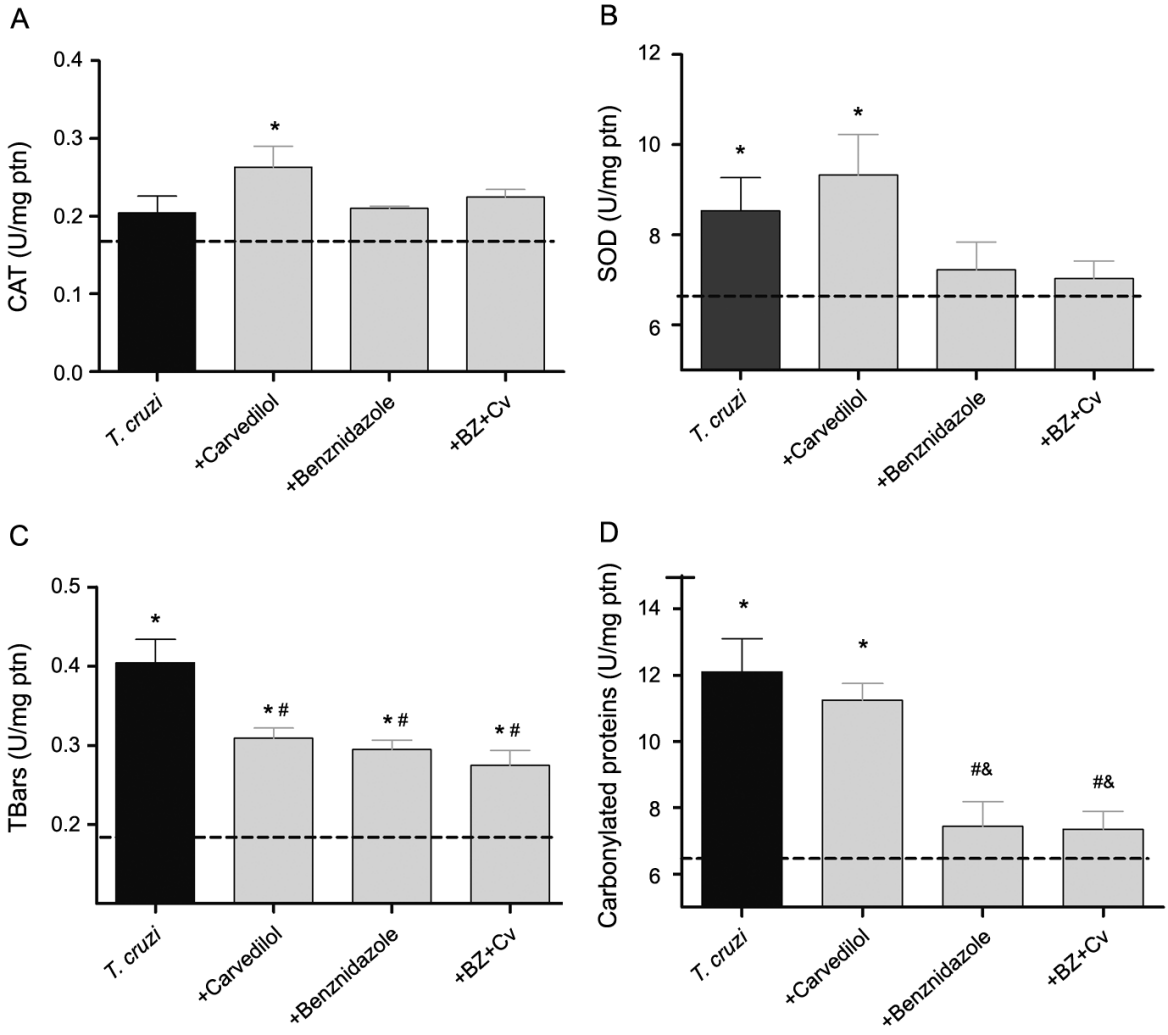
oxidative stress in the cardiac homogenate. Activity of the antioxidant enzymes (A) Catalase (CAT), (B) Superoxide dismutase (SOD), and (C) Thiobarbituric acid reactive substances (TBARS), and (D) Carbonylated proteins (PC) in cardiac tissue of C57BL/6 mice infected with the Colombian strain of *Trypanosoma cruzi*, submitted or not to the proposed to carvedilol (Cv) and/or benznidazole (Bz) therapies. Bars in black represents the group of infected/untreated animals, bars in gray represents the infected groups under different therapeutic strategies. Dashed line represents the serum concentration of these cytokines/chemokines in uninfected animals. * p <0.05 when the uninfected group was compared to the infected groups; # p < 0.05 when the infected group was compared with infected groups treated with different drugs; & p < 0.05 when comparing the treatment with Cv with the other therapeutic strategies.

*Oxidative stress* - In this current study, CAT (Fig. 6A) activity was increased only in the group receiving Cv treatment compared to the control infect group and to the groups treated with Bz and Bz + Cv. Regarding the SOD activity (Fig. 6B) and the oxidative damage markers, when analysing the data concerning carbonylated proteins (Fig. 6D), we observed that the untreated group and the ones receiving Cv in monotherapy presented a higher index of protein oxidative damages, showing statistical differences when compared to infected groups treated with Bz, with the association (Bz + Cv) and with the control group. A different profile was observed when analysing TBARS levels (Fig. 6C) among the groups, since we observed that there were no statistical differences between the infected groups that received any of the proposed therapeutic interventions. However, the groups receiving the proposed treatments differed from those belonging to the control group.

## DISCUSSION

The existing limitations in Chagas chemotherapy prompt various researchers throughout the world to the search of effective and safer therapy alternatives. The inflammatory status provided by *T. cruzi* has been pointed out as the foremost factor modulating this illness in both acute and chronic phases of Chagas disease.^3^ However, the inflammatory panel observed in the acute phase of infection might predict part of the clinical changes and the progression toward chronic heart failure. Among chagasics displaying clinical manifestations, 94.5% of the cases are affected by cardiac complications, where 56% develops heart failure and 38.5% may die of sudden death.^10^ Machado and colleagues^11^ have shown that cardiomyocytes are a potential cellular source of cytokines, chemokines and nitric oxide *in vivo*.^11^ That said, the predominance of an inflammatory environment during the acute phase of this disease might be associated to its symptomatic forms. So, alongside the investigation of new antiparasitological compounds, some drugs have been evaluated in experimental models of *T. cruzi* infection with a potential application in immunomodulation of heart pathology.^8^

Drugs, such as the adrenergic antagonist Cv, primarily intended in cardiovascular application, despite its broadly discussed effects displaying antioxidant activity and improving cardiac damages, such as heart failure, hypertension and thrombosis. It additionally presents an immunomodulatory effect as shown in previous studies.^7^ ^,^ ^12^ In particular, our group has recently observed that Cv therapy in mice infected with the VL-10 strain of *T. cruzi* was capable to reduce the plasma levels of the CCL2 chemokine and to elevate the levels of IL-10 reflecting in the reduction of the inflammatory process in the cardiac tissue.^13^ However, opposing our expectancies, the current results using the inflammatory strain of the *T. cruzi* (Colombian strain) implied that Cv therapy worsened *T. cruzi* infection course in the murine model, enhancing mortality rate, parasitaemia, and leukocyte infiltration as well as increasing the circulating levels of inflammatory chemokines.

Previous study has already shown the capacity of Cv in reducing TNF serum levels, suggesting clinical prognosis improvements in patients holding cardiopathy due to other etiologies.^14^ The given result supports these previous researchers, showing that this therapeutic strategy reduced TNF levels after Cv administration. However, it is known that TNF, as well as IL-12 and IFN-γ, are indispensable cytokines for the parasite control in the acute phase of this illness, especially by the activation of mononuclear cells.^15^ ^,^ ^16^ The TNF blockage demonstrated how vulnerable these animals can become by this infection, reinforcing the importance of this inflammatory mediator in controlling its replication.^17^

In our study, it is important to highlight, that higher mortality rate and parasitaemia levels were also observed in the groups that received the Cv monotherapy. There are few studies in literature concerning the administration of Cv in experimental models. These previous surveys have already shown that Cv is able to increase the survival rate of Syrian hamsters, infected by the *T. cruzi* Y strain, in an acute phase of infection;^18^ studies using other beta-blockers (eg., atenolol and propranolol) have also shown an increase in the survival rate of Swiss and Balb/c mice also infected by the *T. cruzi* Y strain, as well as showing a reduction of parasitism in the cardiac tissue.^19^ ^,^ ^20^ More studies concerning this disease, and Cv treatment are needed for better conclusions. But the obtained data, suggests that the lack of TNF in animals treated with Cv possibly displayed the higher parasitaemia levels in this group and, moreover led to the higher mortality rate, due to its pivotal role in parasite replication.

Additionally, correlating the role of inflammation with the *T. cruzi* pathological mechanisms still faces crucial challenges, since its intensity may be modulated regarding parasite and host’s genetic diversity, and thus steering the course and evolution of the disease. The use of murine models is widely known in *T. cruzi* infection surveys; however, different lineages tend to display distinct immune responses according to parasite’s strain, suggesting genetic regulation engaging host’s resistance. Likewise, this protozoan has a wide intraspecific genetic diversity, currently classified into six DTUs. As stated, the Colombian strain of *T. cruzi* exploited for this trial, displays cardiac tropism and belongs to *T. cruzi* I group (DTU I). Possibly, the intrinsic genetic features of the given strain allied to host’s immune response exerted great influence on the course of the exhibited infection.^8^ ^,^ ^21^

Moreover, the absence of regulatory factors shown by the non-altering levels of IL-10 plasma concentrations, in addition to stimulus persistence induced by the high levels of circulating parasites in groups receiving carvedilol, possibly triggered the increase of the inflammatory chemokines CCL2 and CCL5. Both CCL5/RANTES and CCL2/MCP1 have been implicated in *T. cruzi* infection to the augmented leukocyte infiltration into the inflammatory foci, aiming parasite eradication.^3^ ^,^ ^16^ Studies, such as the ones carried by Machado and colleagues^22^ and Hardison and colleagues^23^ showed that mice lacking CCL5 receptor (CCR5) are more susceptible to *T. cruzi* infection because of the reduction of cell migration into the infected tissues.^22^ ^,^ ^23^ The CCL2 in high concentrations was previously described in the serum of chagasic patients and associated to different levels of heart dysfunction.^24^ Accordingly, the increase in parasite burden in C57BL/6 serum linked to the beta-blocker therapy, possibly, behaved as stimuli for its greater production, enhancing intense leukocyte recruitment in order to eliminate this harmful agent. Another interesting aspect, is that its high plasma levels was already shown in previous signs of myocardial dysfunction, leading to myocarditis through the retention of leukocytes at the infection site, which could later steer to cardiac injuries.^25^ Also, despite the strain’s cardiac tropism, the massive leukocyte infiltration verified in this group might be linked to these chemokine’s high concentrations, reinforcing previous findings that showed that high concentrations of CCL2 might lead to intense and focal inflammatory infiltration in this murine model, highlighting our current research.^26^

In spite of this, the above explanation is not consistent when analysing the oxidative stress data. Studies have shown that Cv has antioxidant properties and this is mainly due to its carbazole structure, and the hydroxylated metabolites in its molecule.^27^ These metabolites give this beta-blocker an even greater antioxidant capacity when compared to Cv itself.^28^ While observing the TBARS marker, it is possible to note that it was reduced by Cv administration, a similar pattern is observed in the groups receiving Bz and the combination therapy matching the normal animals. The antioxidant potential of this drug may be associated with its ability to bind to Fe (III) and Cu (II), and by that avoiding lipid and protein oxidation mediated by these transition metals, and this could possibly explain the decrease of the lipid peroxidation biomarker (TBARS). However, Cv did not show activity on the concentration of carbonylated proteins, as well as in the production of the antioxidant enzymes SOD and CAT. Assuming this interference of the Cv on the oxidative enzymes, this new environmental post treatment might have contributed to the growth of parasites in the initial phase of the infection. This hypothesis can be supported by previous studies demonstrating that ROS products can be associated since to the elimination than to the *T. cruzi* growth.^29^ ^,^ ^30^ These results are in contrast with those found by Budni and colleagues,^28^ where Cv was able to increase the production of these antioxidants enzymes in chronic chagasic patients, but it is important to underline that inflammatory response in this illness is carried out differently depending on the host genetics, strain, and inoculum utilised.^28^

*In conclusion* - Indeed, the Cv and Bz monotherapies administered in the initial phase of the *T. cruzi* infection displayed interference towards inflammatory response. Even in the face of the immunomodulatory actions induced by Bz, our data suggests that the Cv, in this particular model using the Colombian strain of *T. cruzi*, might cause damage the host, hypothesising that the reduction of the TNF marker, concomitant with the increase of CCL2, CCL5 and CAT appears to have contributed to higher inflammation in heart tissue, as well as to the concomitant increase of circulating parasites and consequently the lower survival rate observed. In this way, the combination (Cv + Bz) was not efficient since the most observed immunomodulatory actions were driven by the Bz in this proposed experimental infection design. We assume that further investigation is still necessary to evaluate the Cv whether this therapy would be more promising at an early or a later stage of infection and, most importantly, to understand how the genetic background of this parasites can influence the pleiotropic action of this beta-blocker towards *T. cruzi* infection.
